# Hypocomplementemic Urticarial Vasculitis Associated With Hashimoto’s Thyroiditis and Hepatitis B Virus Infection: A Case Report

**DOI:** 10.7759/cureus.29643

**Published:** 2022-09-27

**Authors:** Lucian G Scurtu, Mariana Costache, Daniela Opriș-Belinski, Olga Simionescu

**Affiliations:** 1 Department of Dermatology I, Colentina Clinical Hospital, Carol Davila University of Medicine and Pharmacy, Bucharest, ROU; 2 Department of Pathology, Bucharest University Emergency Hospital, Bucharest, ROU; 3 Department of Pathology, Carol Davila University of Medicine and Pharmacy, Bucharest, ROU; 4 Department of Rheumatology and Internal Medicine, “Sfanta Maria” Clinical Hospital, Carol Davila University of Medicine and Pharmacy, Bucharest, ROU

**Keywords:** arthritis, dapsone, leukocytoclasis, vasculitis, urticaria, hypocomplementemia

## Abstract

Urticarial vasculitis (UV) is an uncommon condition characterized by recurrent episodes of urticarial lesions and angioedema and the pathological features of leukocytoclastic vasculitis. UV divides into two subgroups based on the level of serum complement. Usually, patients with hypocomplementemia experience internal organ involvement and an unfavorable prognosis. We report the case of a 33-year-old woman with a history of hepatitis B infection and autoimmune thyroiditis who developed hypocomplementemic urticarial vasculitis with recurrent angioedema and arthralgia. Complete remission was achieved using dapsone in monotherapy. We suggest dapsone as a potential treatment of choice for hypocomplementemic urticarial vasculitis. This clinical case emphasizes the need for urticarial vasculitis treatment guidelines.

## Introduction

Urticarial vasculitis (UV) is a relatively rare disorder (3% to 20% in chronic urticaria patients) that associates recurrent episodes of urticarial lesions and angioedema and the histopathological features of leukocytoclastic vasculitis. Individual lesions usually persist longer than 24 hours and resolve with residual hyperpigmentation. UV is idiopathic in most patients but can also occur in the context of infections, connective tissue disorders, drug reactions, Schnitzler syndrome, or paraneoplastic syndrome [1,2].

UV divides into two subgroups based on the level of serum complement: normocomplementemic UV (NUV) and hypocomplementemic UV (HUV). Patients with NUV display a better prognosis and usually have an absent or mild systemic involvement [1]. There is no consensus on a universally beneficial treatment and treatment guidelines are lacking [3]. This paper describes the case of a 33-year-old woman with a history of hepatitis B infection and autoimmune thyroiditis who develops HUV with recurrent angioedema.

## Case presentation

A 33-year-old female patient was referred to our dermatology clinic, with an eight-month history of recurrent urticaria and numerous presentations to the emergency room with angioedema. She had been experiencing diffuse arthralgia three months prior to the presentation. During the physical examination, she presented pale, pruritic, disseminated urticarial plaques, distributed on the back and upper limbs. No dysphagia or tongue edema was noticed. Initial investigation showed a complete blood count within normal limits. The patient was an inactive hepatitis B carrier with undetectable viral load (HBV DNA), positive antibodies to HVB core antigen (anti-HBc), and HBV surface antigen (HBsAg). More laboratory examinations revealed positive anti-thyroglobulin antibodies (596 IU/mL; normal range < 115 IU/mL) and anti-thyroid peroxidase antibodies (19.96 IU/mL; normal range < 5.61 IU/mL), with normal thyroid-stimulating hormone (TSH), triiodothyronine (T3), and free-thyroxine (T4), suggestive for autoimmune (Hashimoto’s) thyroiditis with euthyroidism. The patient did not present any family history of autoimmune thyroiditis or connective tissue disorders. 

**Figure 1:**
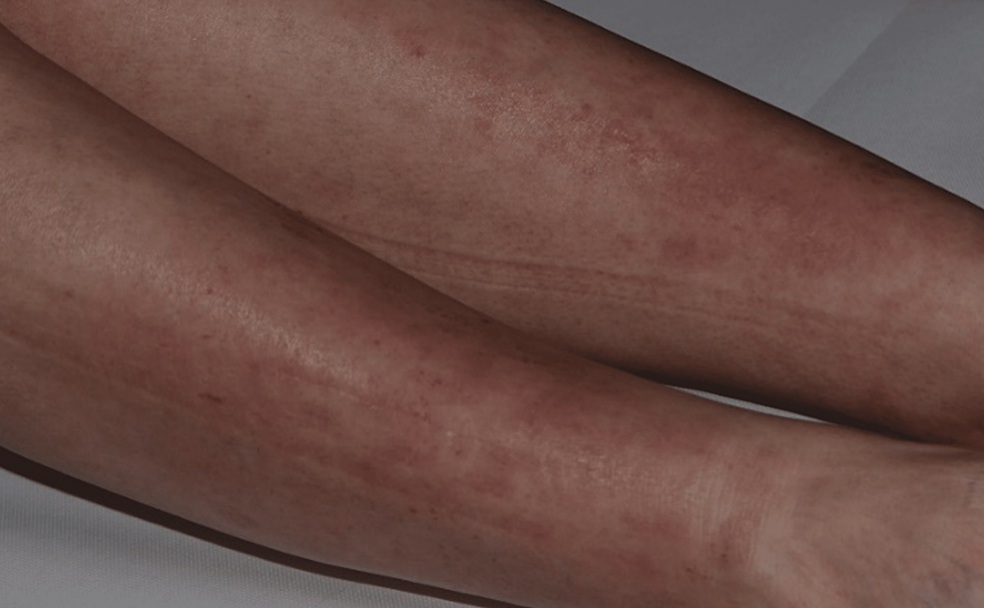
Livedo reticularis on the lower limbs, with a purplish, net-like appearance.

**Figure 2:**
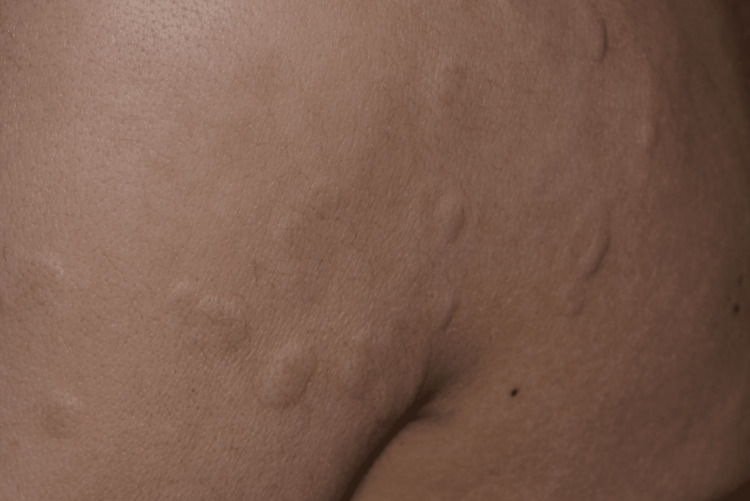
Annular, edematous papules on the patient’s back were suggestive of cutaneous mast cell disorder.

The patient was referred to an endocrinologist and received a combination of antihistamine drugs and topical steroids, but afterward was lost to follow-up until six months later. She was then admitted to our department with intense arthralgia involving the knees, wrists, and interphalangeal joints of the hands. She presented livedo reticularis on the inferior limbs, and scattered, annular, edematous papules on the back and anterior thorax, suggesting a mast cell or connective tissue disorder (Figure 1 and Figure 2). The oral mucosa, soles, palms, and scalp were unaffected. The patient reported no fever.

Laboratory examinations showed low: C3 level (40mg/dL, normal value: 90 to 180 mg/dL), C4 level (12 mg/dL, normal value: 10 to 40 mg/dL) and C1q level (2.8 mg/dL, normal value: 5 to 25 mg/dL). Level of anti-C1q antibodies was 16.3 ug/mL (normal value < 4.0 ug/mL). Cryoglobulins, anti-nuclear antibodies, anti-double-stranded DNA antibodies, anti-U1-ribonucleoprotein anti-bodies, anti-SSA (Sjogren’s syndrome type A antigen) antibodies, anti-SSB (Sjogren’s syndrome type B antigen) antibodies, and anti-Smith antibodies were not detected. The erythrocyte sedimentation rate was 45 mm/h (normal value < 20 mm/h). The complete blood count and urinalysis showed no anomalies.

**Figure 3:**
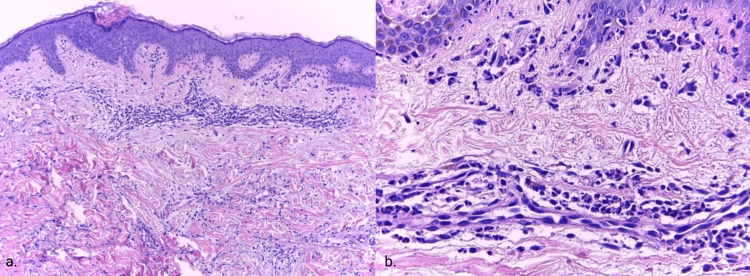
Histopathological examination a. dermal inflammation (“a busy dermis”), with dense perivascular inflammatory infiltrates (HE, 40x). b. magnified vision (200x) reveals an inflammatory infiltrate with neutrophils, lymphocytes, histiocytes, and rare eosinophils, and neutrophil degeneration (leukocytoclasis) with nuclear dust (HE) HE: hematoxylin-eosin

A cutaneous punch biopsy was performed, and hematoxylin-eosin (HE) analysis showed lymphocytic dermal inflammation, with a less prominent neutrophilic component and leukocytoclastic vasculitis of the blood vessels, consistent with UV (Figure 3). Other special stains (such as toluidine blue) were inconclusive. Mast cell disorders were excluded. The patient was subsequently diagnosed with HUV. After a negative result for erythrocytic glucose-6-phosphate dehydrogenase deficiency, daily administration of 75 mg dapsone was started, with rapid improvement after the first two weeks. Dapsone dosage was gradually tapered to 50 mg per day, for 14 months. No adverse effects occurred. After treatment completion, the patient’s physical examination showed no skin lesions or arthralgia and C3 level was normal (92 mg/dL).

## Discussion

First described by Mc Duffie et al in 1973 [4], UV is clinically associated with urticaria. Because of the complement classical pathway activation and vessel inflammation, it is histologically different from urticaria. Based on complement levels and involvement of internal organs, there are three types of UV: NUV, HUV, and HUV syndrome (HUVS). Usually, NUV is benign and self-limited, while HUV and particularly HUVS involve internal organs. Women with UV are eight times more frequently affected than men. The median age at presentation for these patients' involvement is 48 years. However, we consider that our patient's two comorbidities have triggered this earlier debut. The presence of chronic, urticarial lesions, particularly affecting the upper body, persisting for more than 24 hours, represents the main manifestation of HUV. Fifty percent of these patients experience arthralgia and angioedema, with lips, tongue, and periorbital soft tissue involvement, explained by a more profound involvement of the vessels [3,5].

HUV represents an immune-complex mediated vasculitis, produced via a type III hypersensitivity reaction. The vessels’ damage mechanisms involve immune complexes formation with complement system activation, anti-C1q antibodies, and lymphocytes T response. However, anti-C1-q antibodies are not specific to HUV and may also be positive in systemic lupus erythematosus, Goodpasture syndrome, rheumatoid arthritis, systemic sclerosis, and Sjögren syndrome [5,6]. Positive C1-q antibodies are associated with a poorer prognosis and systemic involvement [7], but our patient experienced a mild evolution and no internal organs were involved.

Diagnostic criteria for HUVS consist of two major criteria (hypocomplementemia and recurrent urticaria lasting more than six months) and a minimum of two minor criteria (vasculitis on skin biopsy, arthralgias or arthritis, glomerulonephritis, ocular inflammation, abdominal pain, and positive anti-C1q antibodies with decreased C1q level). Elevated HBsAg is an exclusion criterion for HUVS, but not for HUV [5,8]. We have identified the hepatitis B virus as one of the triggers for our patient’s UV. From this point of view, we have classified this patient with HUV rather than HUVS. Additionally, the patient’s optimistic evolution has endorsed this diagnosis.

A retrospective study proved that only 4% of the patients with chronic spontaneous urticaria presented elevated anti-thyroglobulin and anti-thyroid peroxidase antibodies while 41,7% of the patients with UV presented these antibodies above the reference range [9]. Therefore, we consider that autoimmune thyroiditis could be a second trigger for our patient’s disease. Complete and partial cutaneous remission in HUV can be achieved using corticosteroids, dapsone, and H1-antihistamines. Additionally, treatments with hydroxychloroquine, colchicine, and indomethacin may lead to some modest results [10]. The patient experienced clinical remission with dapsone, thus avoiding the adverse effects of long-term oral corticosteroid use.

## Conclusions

UV should be considered in patients with recurrent urticaria and frequent episodes of angioedema. UV classifications are of significant importance in clinical practice and complement levels should be invariably tested in these patients. Dapsone can be regarded as a potential treatment of choice for HUV, especially in patients with mild systemic involvement. This clinical case may support physicians treating patients with persistent urticarial exanthemas and raises awareness of the necessity of UV standardized treatment guidelines. 
